# Effectiveness and safety of dupilumab in patients with chronic rhinosinusitis with nasal polyps and associated comorbidities: a multicentric prospective study in real life

**DOI:** 10.1186/s12948-022-00171-2

**Published:** 2022-05-19

**Authors:** Eustachio Nettis, Luisa Brussino, Vincenzo Patella, Laura Bonzano, Aikaterini Detoraki, Elisabetta Di Leo, Maria Maddalena Sirufo, Cristiano Caruso, Fabio Lodi Rizzini, Mariaelisabetta Conte, Mona-Rita Yacoub, Massimo Triggiani, Erminia Ridolo, Luigi Macchia, Giovanni Rolla, Raffaele Brancaccio, Amato De Paulis, Giuseppe Spadaro, Danilo Di Bona, Angela Maria D’Uggento, Lia Ginaldi, Francesco Gaeta, Eleonora Nucera, Kliljeda Jaubashi, Danilo Villalta, Lorenzo Dagna, Domenico Ciotta, Francesco Pucciarini, Diego Bagnasco, Giorgio Celi, Fulvia Chieco Bianchi, Lorenzo Cosmi, Maria Teresa Costantino, Maria Angiola Crivellaro, Simona D’Alò, Pietro del Biondo, Stefano Del Giacco, Mario Di Gioacchino, Linda Di Pietro, Elisabetta Favero, Sebastiano Gangemi, Gabriella Guarnieri, Enrico Heffler, Maria Stefania Leto Barone, Carla Lombardo, Francesca Losa, Andrea Matucci, Paola Lucia Minciullo, Paola Parronchi, Giovanni Passalacqua, Stefano Pucci, Oliviero Rossi, Lorenzo Salvati, Michele Schiappoli, Gianenrico Senna, Andrea Vianello, Alessandra Vultaggio, Yang Baoran, Cristoforo Incorvaia, Giorgio Walter Canonica

**Affiliations:** 1 Department of Emergency and Organ Transplantation, School of Allergology and Clinical Immunology, University of Bari Aldo Moro, Policlinico Di Bari, Bari, Italy; 2 Department of Medical Sciences, University of Turin, Corso Achille Mario Dogliotti, 14, 10126 Turin, Italy; 3 S.S.D.D.U. Allergologia E Immunologia Clinica, AO Ordine Mauriziano Umberto I, 10128 Turin, Italy; 4 Division of Allergy and Clinical Immunology, Department of Medicine ASL Salerno, Santa Maria Della Speranza Hospital, Salerno, Italy; 5 Dermatology Unit, Azienda USL-IRCCS di Reggio Emilia, Reggio Emilia, Italy; 6 Department of Internal Medicine, Clinical Immunology, Clinical Pathology and Infectious Disease, Azienda Ospedaliera Universitaria Federico II, Napoli, Italy; 7 Section of Allergy and Clinical Immunology, Unit of Internal Medicine, “F. Miulli” Hospital, Strada Provinciale per Santeramo Km 4.100, Acquaviva delle Fonti, Bari, Italy; 8 Department of Life, Health and Environmental Sciences, University of L’Aquila, 67100 L’Aquila, Italy; 9 Dipartimento di Scienze Mediche e Chirurgiche, Digestive Disease Center, Fondazione Policlinico A. Gemelli, IRCCS, Università Cattolica del Sacro Cuore, Rome, Italy; 10 Facoltà Medicina e Chirurgia, Università Studi Brescia, SSVD Allergologia - Spedali Civili Brescia, Brescia, Italy; 11 Struttura Dipartimentale di Immunologia ed Allergologia Azienda Sanitaria Friuli Occidentale, Presidio Ospedaliero di Pordenone, Pordenone, Italy; 12 Unit of Immunology, Rheumatology, Allergy and Rare Diseases, IRCCS San Raffaele Scientific Institute, Milan, Italy; 13 Division of Allergy and Clinical Immunology, University of Salerno, Fisciano, Italy; 14 Department of Medicine and Surgery, University of Parma, Parma, Italy; 15 Dipartimento di Scienze Mediche, Università Di Torino, Turin, Italy; 16 Department of Economics and Finance, University of Bari, Bari, Italy; 17 Allergy and Clinical Immunology Unit, AUSL 04, 64100 Teramo, Italy; 18 Clinica Delle Malattie Respiratorie e Allergologia Dipartimento di Medicina Interna (DIMI), Università Degli Studi di Genova IRCCS Ospedale Policlinico San Martino, Genova, Italy; 19 UO Allergologia e Immunologia Clinica, ASST Mantova, Mantova, Italy; 20 UOC di Fisiopatologia Respiratoria, Azienda Ospedale-Università di Padova, Padova, Italy; 21 Dipartimento di Medicina Sperimentale e Clinica, Università Degli Studi di Firenze, Florence, Italy; 22 Occupational Health Unit and AllergologyPadova University Hospital, Department of Cardiac Thoracic Vascular Sciences and Public Health, University of Padova, 35128 Padova, Italy; 23 UOC Allergologia, Ospedale di Civitanova Marche, ASUR Marche Area Vasta 3, Civitanova Marche, Italy; 24 Scuola di Specializzazione in Allergologia ed Immunologia Clinica, Università Degli Studi “Gabriele d’Annunzio” di Chieti-Pescara, Chieti, Italy; 25 Allergologia e Immunologia Clinica - Dipartimento di Scienze Mediche e Sanità Pubblica, Università Degli Studi Di Cagliari, Cagliari, Italy; 26 Center of Advanced Studies and Technology, G. d’Annunzio University, Chieti, Italy; 27 YdA - Institute for Clinical Immunotherapy and Advanced Biological Treatments, Pescara, Italy; 28 Centro Allergologico e Malattie Rare, Dipartimento di Medicina Ospedale Ca’ Foncello, Treviso, Italy; 29 Department of Clinical and Experimental Medicine, School and Operative Unit of Allergy and Clinical Immunology, University of Messina, 98124 Messina, Italy; 30 Department of Cardiac Thoracic Vascular Science and Public Health, University of Padova, Padova, Italy; 31 Personalized Medicine, Asthma and Allergy, IRCCS Humanitas Research Hospital, Rozzano, MI Italy; 32 Department of Biomedical Sciences, Humanitas University, Pieve Emanuele, MI Italy; 33 UF Riabilitazione Respiratoria, Casa di Cure Villa Serena, Palermo, Italy; 34 Allergy Unit, Villa Igea Hospital. A.P.S.S. Trento, Trento, Italy; 35 Immunoallergology Unit, Careggi University Hospital, Florence, Italy; 36 Immunology and Cell Therapy Unit, Careggi University Hospital, Florence, Italy; 37 UOC Allergologia E Asma Center, Azienda Ospedaliera Universitaria Integrata di Verona, Verona, Italy; 38 Private Practice, Milan, Italy

## Abstract

**Background:**

Biologics are currently one of the main treatment options for a number of diseases. The IgG4 monoclonal antibody dupilumab targets the Interleukin-4 receptor alpha chain, thus preventing the biological effects of the cytokines IL-4 and IL-13, that are essential for the Th2 response. Several controlled trials showed that dupilumab is effective and safe in patients with atopic dermatitis (AD), severe asthma and chronic rhinosinusitis with nasal polyps (CRSwNP), thus resulting in approval by regulatory agencies. Aim of the study was to evaluate the efficacy and safety of dupilumab in adult patients with CRSwNP stratified by common overlapping comorbid conditions.

**Methods:**

We performed a multicenter, observational, prospective study enrolling adult patients with severe CRSwNP who had started dupilumab treatment in the context of standard care from January 2021 to October 2021. Data were collected from twentynine Italian secondary care centers for allergy and clinical immunology, all of which were part of the Italian Society of Allergy, Asthma and Clinical Immunology (SIAAIC). A number of efficacy parameters were used. Patient data were compared using the Wilcoxon test for paired data. All statistical analyses were performed with SPSS version 20 (IBM, Armonk, NY, USA).

**Results:**

In total, 82 patients with nasal polyposis were identified. A significant improvement was detected for all the applied efficacy parameters, i.e. 22-item Sino-Nasal Outcome Test (SNOT-22) and bilateral endoscopic nasal polyp score (NPS) scores for CRSwNP, Rhinitis Control Scoring System (RCSS) and Rhinoconjunctivitis Quality of Life Questionnaire (RQLQ) scores for allergic perennial rhinitis, Forced Expiratory Volume in the 1st second (FEV1) and Asthma Quality of Life Questionnaire (AQLQ) scores for asthma, Eczema Area and Severity Index (EASI) and Dermatology Life Quality Index (DLQI) scores for AD. A non-significant improvement was also obtained in the Urticaria Activity Score over 7 days (UAS7) for chronic spontaneous urticaria. Treatment with dupilumab was well tolerated.

**Conclusions:**

These data suggest that dupilumab treatment in patients suffering from CRSwNP and associated comorbidities may be suitable. Such outcome, although confirmation by trials is warranted, suggests the possibility to treat different disorders with a single therapy, with favorable effects especially under the cost-effectiveness aspect.

## Introduction

Although the term biological also encompasses therapies that have been in use for decades, such as vaccines, blood and its component, cells, allergens, genes, a modern classification defines biologics as the product obtained from living organisms or containing components of living organisms. Another key feature is the protein content of biologics that control the action of other proteins and cellular processes, genes encoding for vital proteins, or cells that produce substances suppressing or activating components of the immune system [1]. The number of products derived from human, animal, or microorganisms by using biotechnology is continuously expanding. As far as upper airways diseases are concerned, chronic rhinosinusitis with nasal polyps (CRSwNP) has long been considered to be closely related to aspirin sensitization [2], but today the crucial role of cytokines in the pathogenesis of this disease is also acknowledged, notably Th2 cytokines due to their role in eosinophils recruitment and maintenance of inflammatory response [3].

In the latest years, a number of monoclonal antibodies (including mepolizumab, reslizumab, and benralizumab) were approved to treat, in addition to severe asthma, other disorders linked to type 2 immune responses including CRSwNP [4]. Dupilumab is a IgG4 monoclonal antibody which targets the IL-4 receptor alpha chain (IL-4Rα), thus inhibiting the biological effects of the cytokines IL-4 and IL-13, that are crucial for the Th2 response [5]. Dupilumab was demonstrated by meta-analyses to be effective and safe also for the treatment of adult patients with moderate-to-severe atopic dermatitis (AD) not adequately controlled with standard treatment [6] as well as for severe CRSwNP [4] and uncontrolled asthma [7].

The objective of this study is intended to describe effectiveness of dupilumab in the real-world setting and in the management of patients with CRSwNP stratified by common overlapping comorbid conditions.

## Materials and methods

### Study design and patients

We performed a multicenter, observational, prospective study enrolling adult patients with severe CRSwNP who had started dupilumab treatment in the context of standard care from January 2021 to October 2021. Data were collected from twenty-nine Italian secondary care centers for allergy and clinical immunology, all of which were part of the Italian Society of Allergy, Asthma and Clinical Immunology (SIAAIC). To participate in the study, each center was asked to provide data from patients ≥ 18 years of age with CRSwNP who had undergone previous treatment with systemic corticosteroids (SCS), had a contraindication or intolerance to SCS in the previous 2 years, or had a history of sinonasal surgery.

At screening, patients must have NPS of at least 1 (maximum 8), and exhibit at least two of the following symptoms: nasal congestion or obstruction and either loss of smell or nasal discharge (anterior or posterior). Patients were required to have not undergone previous nasal polyp removal surgery within 6 months before screening. Use of monoclonal antibodies, immunosuppressants, or anti-IgE therapy was not allowed from 2 months before treatment until the end of the study. Patients who met the inclusion criteria underwent complete ear, nose, and throat examination.

All procedures complied with the Helsinki Declaration of 1964, revised in 2013. The study protocol was approved by the ethical committee of Naples University Hospital, Italy. Informed consent was obtained from all patients who agreed to participate to this study.

At baseline, all patients received a loading dose of dupilumab 300 mg subcutaneously administered by a clinician, followed by dupilumab 300 mg every other week for 16 weeks. A wash-out period was not required. Patients were asked to discontinue systemic immune-suppressants before starting dupilumab treatment. Rescue treatment could be provided to patients at the investigator’s discretion. Throughout the study period, patients were required to maintain their pre-treatment therapy for the management of CRSwNP (i.e., saline nasal lavage, systemic antibiotics) and other comorbidities.

Patients were assessed for medical history, demographics, comorbid diseases (ie, allergic rhino-conjunctivitis, asthma, atopic dermatitis, chronic spontaneous urticaria), concomitant medications or procedures, adverse events, and efficacy outcomes at baseline; and efficacy every 4 weeks (weeks 4–16).

Drug safety was evaluated by recording and monitoring the frequency and severity of treatment-emergent and serious adverse events.

### Procedures, outcomes and statistical analysis

In CRSwNP patients, outcome measures at baseline and after 4 months included endoscopic NPS as assessed by endoscopy for each nostril separately and graded by polyp size (range: 0 to 4); 22-item Sino-Nasal Outcome Test (SNOT-22) (range: 0–110) which assesses the quality of life and the symptoms of sinusitis; patient reported total symptom score (TSS) (range 0–9), in which patients recorded severity of symptoms (nasal congestion [NC], loss of smell [LoS], and anterior/posterior rhinorrhea) in a daily symptom e-diary using a 4-point scale (0 = no symptoms; 1 = mild symptoms; 2 = moderate symptoms; and 3 = severe symptoms) and calculated as a composite severity score consisting of the sum of the NC (range 0–3), LoS (range 0–3), and rhinorrhea (average of anterior/posterior scores for nasal discharge) (range 0–3) symptom scores. Rhinosinusitis disease and smell capacity were also documented according to “the visual analog method”[8]. The intensity of every symptom was assessed on the Visual Analogue Scale (VAS) from 0 to 10.

The comorbidities were evaluated using: Rhinitis Control Scoring System (RCSS) (range: 10–50), Rhinoconjunctivitis Quality of Life Questionnaire (RQLQ) (range: 0–6), Spirometry, Asthma Control Test (ACT) (range: 0–25), Asthma Quality of Life Questionnaire (standardized version) (AQLQ[S]) (range: 0–7), Eczema Area and Severity Index (EASI) score (range: 0–72); peak score on the Numerical Rating Scale (NRS) for pruritus (range: 0–10), peak score on the NRS for sleep (range: 0–10), Dermatology Life Quality Index (DLQI) (range: 0–28), Urticaria Activity Score over 7 days (UAS7) (range 0–42).

Within-patient improvement in SNOT-22, RQLQ, ACT and AQLQ(S) scores of at least 8.9, 0.5, 3 or 0.5 respectively, was considered clinically meaningful, as defined by the questionnaire developers [9–12]. Controlled asthma was defined as asthma with a composite ACT score of more than 19, and the absence of exacerbations during the 16-week treatment period [10].

Skin prick tests using commonly available foods and inhalants were performed according to the established guidelines on all patients. Clinical evaluation also included total serum IgE levels and the blood eosinophil count. Total serum IgE levels were measured using immunofluorometric assay and expressed in KUA/L, according to the manufacturer’s instructions. Total IgE normal values were considered to be < 100 KU/L. An eosinophil count < 500/mm^3^ was considered normal.

Patient data were compared using the Wilcoxon test for paired data. All statistical analyses were performed with SPSS version 20 (IBM, Armonk, NY, USA). The threshold for statistical significance was set at P < 0.01.

## Results

In total, 82 patients with nasal polyposis were identified. Eight patients were excluded in the analysis due to the following reasons: three patients stopped treatment before 16 weeks, but none was due to treatment failure (2 stopped due to gastrointestinal symptoms unrelated to dupilumab administration, 1 due to personal reasons), and the remaining 5 had missing baseline data. Seventy-four patients with CRSwNP who received at least one dose of dupilumab at the study centres were included in the study. Baseline demographics and characteristics are shown in Table 1.

**Table 1:** Characteristics of patients included in the study (N = 74)

Variable	Value*
Age (y)	46.5 ± 19.8
Sex, female	32 (43.2)
BMI	25.5 ± 6.1
Nasal polyp duration (y)	8.8 ± 2.0
Nasal polyp surgery	
≥ 1 previous surgery	61 (82.4)
≥ 3 previous surgeries	11 (14.9)
Time since most recent polyp surgery (y)	2.5 ± 4.9
Bilateral endoscopic NPS	5.0 ± 2.0
SNOT-22 total score	54.5 ± 28.8
Nasal congestion or obstruction score	3.0 ± 1.0
Missing, n (%)	5 (6.8)
Loss-of-smell score	3.0 ± 1.0
Missing, n (%)	5 (6.8)
Anterior/posterior rhinorrhea score	2.0 ± 1.0
Missing, n (%)	5 (6.8)
Patient reported total symptom score	8.0 ± 2.0
Missing, n (%)	5 (6.8)
Rhinosinusitis disease severity VAS	9.0 ± 2.8
Missing, n (%)	16 (21.6)
Smell VAS	9.0 ± 2.0
Missing, n (%)	15 (20.3)
NSAIDs intolerance n (%)	24 (32.4)
Positive skin prick test results	50 (67.6)
Allergic rhinitis	46 (62.2)
RCSS score	24.0 ± 12.5
Missing, n (%)	3 (6.5)
RQLQ score	3.7 ± 1.1
Missing, n (%)	3 (6.5)
Allergic conjunctivitis	24 (32.4)
Atopic keratoconjunctivitis	3 (4.0)
Asthma	46 (62.2)
FEV1 (L)	2.7 ± 0.8
Missing, n (%)	2 (4.3)
FEV1 (% predicted)	90.5 ± 15.3
Missing, n (%)	2 (4.3)
ACT score	18.0 ± 6.8
AQLQ (S) score	4.3 ± 2.80
Atopic dermatitis	10 (13.5)
EASI score	34.2 ± 20.0
Peak score on NRS for pruritus	8.5 ± 1.0
Peak score on NRS for sleep	8.0 ± 0.8
DLQI score	24.0 ± 12.5
Food allergy	6 (8.1)
Oral allergy syndrome	1 (1.4)
Urticaria	4 (5.4)
Anaphylactic shock	1 (1.4)
Eosinophilic esophagitis	0 (0)
Chronic spontaneous urticaria	3 (4.1)
UAS7	12.0 ± 4.0
Use of systemic corticosteroids in the preceding 16 weeks	
Maintenance use	50 (67.6)
Median daily dose mg	6.9 ± 9.3
IgE (KUA/L)	451.5 ± 286.8
Missing, n (%)	17 (23)
Eosinophils (cells/mm^3^)	550.0 ± 500.0
Missing, n (%)	13 (17.6)

^*^Data are median ± IQR or n (%)

*ACT* Asthma Control Test, *AD* Atopic Dermatitis, *AQLQ (S)* Asthma Quality of Life Questionnaire (standardized version), *BMI* Body Mass Index, *DLQI* Dermatology Life Quality Index, *EASI* Eczema Area and Severity Index, *FEV1* Forced Expiratory Volume in 1 s, *IQR* Interquartile Range, *NPS* Nasal Polyp Score, *NRS* Numerical Rating Scale, *NSAIDs* Non-steroidal anti-inflammatory drugs, *RCSS* Rhinitis Control Scoring System, *RQLQ* Rhinitis Quality of Life Questionnaire, *SNOT-22* 22-item Sino-Nasal Outcome Test, *UAS7* Urticaria Activity Score, *VAS* Visual Analog Scale

In our cohort, 32 patients (43.2%) were female and the median ± interquartile range (IQR) for patient age was 46.5 ± 19.8 years. The median ± IQR body-mass index was 25.5 ± 6.1 kg/m2. The median ± IQR endoscopic NPS score was 5.0 ± 2.0 while SNOT-22 score was 54.5 ± 28.8. Before enrolment, 50 patients (67.6%) had received systemic glucocorticoids. Fifty patients (67.6%) had positive prick test results. Comorbid type 2/Th2 immune diseases were common.

### Chronic rhinosinusitis with nasal polyps

The endoscopic NPS and total SNOT-22 score significantly diminished from baseline to week 16 (median ± IQR percentage change =  − 50.0 ± 33.3 and median ± IQR percentage change = -54.5 ± 41.6, respectively, p < 0.001) (Table 2), 69 patients (93.2%) achieving at least a 1-point improvement in NPS at week 16. Sixty-eight patients (91.9%) had a clinically meaningful improvement in SNOT-22 score (at least 8.9 points). Improvements in NPS and SNOT-22 occurred as early as the first assessment timepoint after the start of dupilumab treatment (within the first 4 to 8 weeks), with continuous improvement evident up to the end of treatment in the study.

**Table 2:** Change in outcome measures between baseline and 16 weeks for 74 dupilumab-treated patients with CRSwNP

Outcome	Baseline	Week 16	*P*-value *
Bilateral endoscopic NPS (scale 0–8)			
Median ± IQR	5.0 ± 2.0	2.0 ± 2.5	< 0.001
SNOT-22 score (scale 0–110)			
Median ± IQR	54.5 ± 28.8	21.0 ± 26.5	< 0.001
Nasal congestion or obstruction score (scale 0–3)			
Median ± IQR	3.0 ± 1.0	1.0 ± 0	< 0.001
Missing, n (%)	5 (6.8)	5 (6.8)	
Loss-of-smell score (scale 0–3)			
Median ± IQR	3.0 ± 1.0	1.0 ± 2.0	< 0.001
Missing, n (%)	5 (6.8)	5 (6.8)	
Anterior/posterior rhinorrhea score (scale 0–3)			
Median ± IQR	2.0 ± 1.0	1.0 ± 1.0	< 0.001
Missing, n (%)	5 (6.8)	5 (6.8)	
Patient reported total symptom score (scale 0–9)			
Median ± IQR	8.0 ± 2.0	3.0 ± 3.0	< 0.001
Missing, n (%)	5 (6.8)	5 (6.8)	
Rhinosinusitis disease severity (VAS 0–10 cm)			
Median ± IQR	9.0 ± 2.8	2.0 ± 2.8	< 0.001
Missing, n (%)	16 (21.6)	16 (21.6)	
Smell (VAS 0–10 cm)			
Median ± IQR	9.0 ± 2.0	2.0 ± 4.0	< 0.001
Missing, n (%)	15 (20.3)	15 (20.3)	
Total IgE (KUA/L)^†^			
Median ± IQR	451.5 ± 286.817 (23)	210.0 ± 372.0	< 0.001
Missing, n (%)		17 (23)	
Eosinophils (cells/mm3)^‡^			
Median ± IQR	550.0 ± 500.0	481.0 ± 500.0	> 0.05
Missing, n (%)	13 (17.6)	13 (17.6)	

*CRSwNP* chronic rhinosinusitis with nasal polyps, *IQR* Interquartile Range, *NPS* Nasal Polyp Score, *SNOT-22* 22-item Sino-Nasal Outcome Test, *VAS* Visual Analog Scale

^*^Compared using the Wilcoxon test for paired data. The threshold for statistical significance was set at P < 0.01

The median percentage change ± IQR in patients reporting the TSS (− 62.5 ± 30), the median percentage change ± IQR in NC score (− 66.7 ± 16.7), the median ± IQR percentage change in LoS score (− 66.7 ± 66.7) and the median ± IQR percentage change in anterior/posterior rhinorrhea score (− 66.7 ± 58.3) each showed a significant decrease from baseline to week 16 (p < 0.001). Patient scores on the rhinosinusitis disease severity and smell capacity assessed with VAS were 9 and 9, before treatment was commenced. After 16 weeks of treatment, the scores were 2 and 2, respectively (p < 0.001).

The percentage of patients who were receiving daily oral corticosteroids (OCS) significantly decreased from 67.6% to 16.2% (p < 0.001). The median ± IQR prednisone equivalent dose was 6.9 ± 9.3 mg at baseline (in 50 patients) and 4.0 ± 3.7 mg at week 16 (in 12 patients).

The median ± IQR total IgE plasma level, measured in 59 patients, decreased at week 16 with a median ± IQR of 210.0 ± 372.0 compared to 451.5 ± 286.8 before dupilumab initiation (p < 0.001).

Concerning the median ± IQR total blood eosinophil count, measured in 63 patients, no significant differences from baseline were found by week 16 (550.0 cell/mm3 ± 500.0 vs 481.0 cell/mm3 ± 500.0; p > 0.05) (Table 2).

### Associated comorbidities

In our patients, clinical history, ENT and skin prick tests were used for the diagnosis of allergic rhinitis. Thirty-one (41.9%) patients were identified as having perennial allergic rhinitis due to allergens that are present year-round (14 women; 17 men) (Table 2). The median ± IQR percentage change of RCSS global total score and RQLQ score from baseline to week 16 was − 40.0 ± 30.7 and − 41.5 ± 31.5, respectively (p < 0.001). The minimal clinically meaningful difference of 0.5 points or more in RQLQ score was observed in 28 of 31 patients (90.3%).

The diagnosis and assessment of severity of asthma was made in 46 patients (20 women; 26 men) according to the Global Initiative for Asthma (GINA) [13] (Table2). All patients in this subgroup had received asthma medications, primarily inhaled corticosteroids and long-acting β-agonists in the preceding year.

With regard to lung function, prebronchodilator Forced Expiratory Volume in the 1st second (FEV1) had improved from a baseline median of 2.7 ± 0.8 L to 2.8 ± 0.9 L after 16 weeks of treatment with dupilumab (p < 0.001), while the median ± IQR FEV1 (per cent of predicted value before bronchodilation) was 90.5 ± 15.3 at baseline and 96.5 ± 11.5 at week 16 (p < 0.001). At the end of the treatment period, 24 patients improved to 0.1 L or more and 9 patients to more than 0.2 L. Disease control was evaluated using ACT. When compared to the baseline measurement of 18.0 ± 6.8, ACT scores had significantly increased to 24.0 ± 5.0 after 16 weeks (p < 0.001).

Thirty-one out of 46 patients (67.4%) achieved the minimally clinically meaningful difference (at least three points) at the 16-week stage. At baseline, 19 out of 46 patients (41.3%) had conditions that were considered as being under control; 40 out of 46 patients (87%) had controlled asthma at week 16. After 16 weeks of therapy with dupilumab, AQLQ scores had significantly increased from a baseline value of 4.3 ± 2.8 to 6.0 ± 1.3 (p < 0.001). Twenty-nine out of 46 patients (63%) achieved the minimally clinically meaningful difference (at least 0.5 points) in the AQLQ. At baseline, a total of 18 patients (39.1%) had developed at least one severe exacerbation requiring the initiation of SCS during the 4-months period preceding the dupilumab treatment. None of the 46 patients developed at least one severe exacerbation during the treatment period (p < 0.001).

Atopic dermatitis (AD) diagnosis was made in 10 patients (4 women; 6 men) according to the revised Hanifin and Rajka criteria [14, 15].

As to AD, dupilumab significantly improved measures of clinical efficacy and quality of life (QoL) at week 16. The median ± IQR EASI score significantly improved from 34.2 ± 20.0 at baseline to 4.3 ± 1.4 at 16 weeks (p < 0.01). The median ± IQR peak score on NRS for pruritus and for sleep significantly decreased from 8.5 ± 1.0 at baseline to 3.0 ± 1.0 at 16 weeks (p < 0.01) and from 8.0 ± 0.8 at baseline to 1.5 ± 2.5 at 16 weeks (p < 0.01), respectively. Finally, the median ± IQR DLQI score significantly decreased from 24.0 ± 12.5 at baseline to 4.0 ± 1.8 at 16 weeks (p < 0.01) (see Table 3).

**Table 3:** Summary of efficacy outcomes in the subgroup of patients with comorbidities at week 16 (N = 57)

Outcome	Baseline	Week 16	*P*-value*
Perennial allergic rhinitis n = 31			
RCSS score			
Median ± IQR	24.0 ± 13.0	16.0 ± 7.5	< 0.001
RQLQ score			
Median ± IQR	3.8 ± 1.1	2.0 ± 1.4	< 0.001
Bronchial asthma n = 46			
FEV1 (L) before bronchodilation-liters			
Median ± IQR	2.7 ± 0.8	2.8 ± 0.9	< 0.001
Missing, n (%)	2 (4.3)	2 (4.3)	
FEV1% of predicted			
Median ± IQR	90.5 ± 15.3	96.5 ± 11.5	< 0.001
Missing, n (%)	2 (4.3)	2 (4.3)	
ACT score			
Median ± IQR	18.0 ± 6.8	24.0 ± 5.0	< 0.001
AQLQ (S) score			
Median ± IQR	4.3 ± 2.8	6.0 ± 1.3	< 0.001
Atopic dermatitis n = 10			
EASI score			
Median ± IQR	34.2 ± 20.0	4.3 ± 1.4	< 0.01
Peak score on NRS for pruritus			
Median ± IQR	8.5 ± 1.0	3.0 ± 1.0	< 0.01
Peak score on NRS for sleep			
Median ± IQR	8.0 ± 0.8	1.5 ± 2.5	< 0.01
DLQI score			
Median ± IQR	24.0 ± 12.5	4.0 ± 1.8	< 0.01
Chronic spontaneous urticaria n = 3			
UAS7 score			
Median ± IQR	12.0 ± 4.0	0 ± 5.0	> 0.05

ACT, Asthma Control Test; AD, Atopic Dermatitis; AQLQ (S), Asthma Quality of Life Questionnaire (standardized version); DLQI, Dermatology Life Quality Index; EASI, Eczema Area and Severity Index; FEV1, Forced Expiratory Volume in 1 s; IQR, Interquartile Range; NRS, Numerical Rating Scale; RCSS, Rhinitis Control Scoring System; RQLQ, Rhinitis Quality of Life Questionnaire; UAS7, Urticaria Activity Score;

^*^Compared using the Wilcoxon test for paired data. The threshold for statistical significance was set at P < 0.01

At 16 weeks, 8 out of 10 patients (80%) had achieved ≥ 75% improvement from baseline as measured by EASI score (EASI-75).

Chronic spontaneous urticaria (CSU) is defined as the spontaneous occurrence of wheals and/or angioedema for six or more weeks [16, 17]. Three patients with CSU who were being treated with dupilumab had a baseline median ± IQR UAS-7 score of 12.0 ± 4.0 points, which.

became lower after 16 weeks (0 ± 5.0; p > 0.05). At the end of the 16-week study, 2 out of 3 patients (66.7%) reported a complete response to dupilumab treatment (UAS7 score = 0).

## Safety

Overall, instances of adverse events (AEs) were low and did not lead to treatment discontinuation. In our cohort, 33.8% of patients overall reported AEs during treatment. Asthenia, arthralgia and de novo conjunctivitis were the most common AEs (Table 4). No treatment-emergent AEs were reported during the study.

**Table 4:** Adverse events reported by patients receiving dupilumab (N = 74)

Adverse events	N (%)
At least 1 adverse event (AE)	25 (33.8)
Asthenia	6 (8.1)
Arthralgia	5 (6.8)
Conjunctivitis	4 (5.4)
Injection-site reaction	3 (4.1)
Nausea	3 (4.1)
Abdominal pain	2 (2.7)
Headache	2 (2.7)
Diarrhea	1 (1.4)
Dizziness	1 (1.4)
Orofacial HSV reactivation	1 (1.4)
Weight gain	1 (1.4)
Any AE leading to discontinuation of study	0 (0)

HSV *Herpes simplex* virus

## Discussion

This is the first observational study that demonstrates the effectiveness of dupilumab in real-life conditions in patients with perennial allergic rhinitis (PAR) and/or asthma and/or AD and/or CSU associated with CRSwNP.

Since the first randomized, double blind-placebo controlled trial in adults with moderate-to-severe atopic dermatitis in 2014, dupilumab has demonstrated its efficacy and safety also in other diseases such as severe uncontrolled asthma [18] and CRSwNP [19]. Globally, the evidence for efficacy is strong, as proved by a number of meta-analyses [20–22]. Acknowledging that the strict rules of controlled trials do not allow inclusion of the majority of patients seen by doctors in current practice, the performance of real-life studies is spreading. A systematic review and meta-analysis including 22 studies in real life encompassing 3303 atopic dermatitis patients confirmed the efficacy of dupilumab also in common practice [23]. Meta-analyses are also available concerning the safety of treatment. Ou et al. in a meta-analysis including 5 randomized controlled trials found that dupilumab slightly increased the risk of headache, and moderately increased the risk of injection-site reaction and conjunctivitis, but had little effect on other infections in adults with moderate-to-severe atopic dermatitis [24]. In a meta-analysis including 8 randomized controlled trials no side effect was reported, while a significantly decrease in the risk for skin infections and eczema herpeticum was observed [25]. In the metanalysis by Han and co-workers cited above also safety was evaluated, with a reassuring overall picture [20]. A pooled analysis showed that the incidence of side effects was 55.5% in patients receiving dupilumab and 45% in patients receiving placebo, this difference being not significant [26]. The other disorder with clear evidence of efficacy of dupilumab is CRSwNP, which was evaluated in a Cochrane Database Systematic Review including 8 randomized controlled trials encompassing 986 adult patients [4]. The results showed that dupilumab improves disease-specific quality of life compared to placebo and reduces the extent of the disease as measured on a computerised tomography scan. The risk of serious adverse events was not increased in actively treated patients. The same authors performed a new Cochrane Systematic Review one year later, adding two trials and assessing also others biologics. The outcome confirmed the previous observations and suggested that treatment with dupilumab probably also results in a reduction in disease severity and in the number of serious adverse events [4].

The peculiarity of our multicenter, observational, prospective study consists in having also evaluated the effects of dupilumab on other common disorders such as PAR and CSU, whose evidence of effectiveness in a real-world setting is far less studied compared to CRSwNP [27], atopic dermatitis [28] and severe asthma [29].

In a prospective observational study [30], Yakamoto et al. and colleagues reported a favorable response to dupilumab treatment in 21 patients with severe comorbid PAR. In this group, significant improvements were observed in subjective symptoms, QoL scores, face scale and findings of nasal cavity after 12 months of treatment.

Another prospective study [31] reported that dupilumab treatment in 31 patients with comorbid PAR was associated with significant improvements in several patient-reported outcomes, including PAR disease control as measured by an RCSS global score and PAR QoL as assessed by validated, disease-specific tools (RQLQ).

As for CSU, three patients with a history of primary/secondary failure to several agents used for their CSU, including standard and high dose of second-generation H1-antihistamines, omalizumab, methotrexate (only in 2 patients), cyclosporine (only in 2 patients), prednisone (only in 1 patient) and ketotifen (only in 1 patient) were treated with dupilumab (600 mg, followed by 300 mg every other week). Within a few months, these patients were free of clinical manifestations [32, 33].

To the best of our knowledge, no studies are available on the effect of dupilumab in patients with concomitant CRSwNP, PAR, asthma, AD and CSU. We performed a study on 82 patients, using a number of efficacy parameters. Significant improvement both in terms of symptoms and QoL was detected for all parameters, i.e. SNOT-22 and NPS scores for CRSwNP, RCSS and RQLQ scores for PAR, FEV1 and AQLQ scores for asthma, EASI, and DLQI scores for AD. These results validate the key role that IL-4 and IL-13 play in the induction and perpetuation of type 2 immune responses implicated in CRSwNP and atopic comorbidities [34]. Treatment with dupilumab was well tolerated in the present study. The most common AEs were asthenia and arthralgia, which were not related to dupilumab treatment. Limitations of this study include the short follow‐up and lack of control patients. These data suggest that dupilumab treatment in patients suffering from CRSwNP and associated comorbidities may be suitable, especially under the pharmaco-economic aspect.

A recent study [35] evaluated the long-term treatment costs and benefits of dupilumab for the treatment of moderate-to-severe adult AD patients over the course of their lifetime. The results suggested that, based on the increase in quality-adjusted life years (QALYs) achieved with dupilumab relative to supportive care (SC), dupilumab is cost-effective compared with SC across a range of annual maintenance prices in this US adult patient population. Up to now no studies have been carried out on the economic value of dupilumab in the treatment of adult patients with CRSwNP [36], but considering the cost-effectiveness demonstrated for AD treatment, it is reasonable to hypothesize that a single treatment in patients with different diseases could be further profitable. In any case, biologics effectiveness on multiple diseases will modify the restriction of use due to the high costs that had been raised for single diseases, even in the presence of risk of death, as in severe uncontrolled asthma [37].

However, the results we observed must be confirmed by studies on larger patient populations and, in particular, for cost-effectiveness aspect, by specific analyses.

## Data Availability

All data generated or analysed during this study are included in this published article.

## Abbreviations

ACT: Asthma control test
AD: Atopic dermatitis
AEs: Adverse events
AQLQ: Asthma Quality of Life Questionnaire
CRSwNP: Chronic rhinosinusitis with nasal polyps
CSU: Chronic spontaneous urticaria
DLQI: Dermatology Life Quality Index
EASI: Eczema Area and Severity Index
FEV1: Forced Expiratory Volume in the 1st second
GINA: Global Initiative for Asthma
IL-4Rα: IL-4 receptor alpha chain
IQR: Interquartile range
LoS: Loss of smell
NC: Nasal congestion
NRS: Numerical Rating Scale
OCS: Oral corticosteroids
PAR: Perennial allergic rhinitis
QALYs: Quality-adjusted life years
RCSS: Rhinitis Control Scoring System
RQLQ: Rhinoconjunctivitis Quality of Life Questionnaire
SC: Supportive care
SCS: Systemic corticosteroids
SIAAIC: Italian Society of Allergy, Asthma and Clinical Immunology
SNOT-22: 22-Item Sino-Nasal Outcome Test
TSS: Patient reported total symptom score
UAS7: Urticaria Activity Score over 7 days
VAS: Visual Analogue Scale
